# A randomized, double-blind, active placebo-controlled study of efficacy, safety, and durability of repeated vs single subanesthetic ketamine for treatment-resistant depression

**DOI:** 10.1038/s41398-020-00897-0

**Published:** 2020-06-26

**Authors:** Paulo R. Shiroma, Paul Thuras, Joseph Wels, C. Sophia Albott, Christopher Erbes, Susannah Tye, Kelvin O. Lim

**Affiliations:** 1 Geriatric Psychiatrist, Minneapolis VA Health Care System, Mental Health Service Line, Minneapolis, MN USA; 2 Assistant Professor, Department of Psychiatry, University of Minnesota, Minneapolis, MN USA; 3 Statistician/Research Methodologist, Minneapolis VA Health Care System, Mental Health Service Line; and Assistant Professor/Research Associate, Department of Psychiatry, University of Minnesota, Minneapolis, MN USA; 4 Staff Anesthesiologist, Minneapolis VA Health Care System, Mental Health Service Line; and Clinical Instructor, University of Minnesota Medical School, Minneapolis, MN USA; 5 Department of Psychiatry, University of Minnesota, Minneapolis, MN USA; 6 Staff Psychologist, Minneapolis VA Health Care System, Mental Health Service Line; and Associate Professor of Psychiatry, University of Minnesota Medical School, Minneapolis, MN USA; 7 Senior Research Fellow, Queensland Brain Institute, The University of Queensland, Queensland, Australia; and Assistant Professor Psychiatry, Psychology and Pharmacology Translational Neuroscience Laboratory, Mayo Clinic, Rochester, MN USA; 8 Drs. T.J. and Ella M. Arneson Land-Grant Chair in Human Behavior, Professor of Psychiatry, Vice Chair for Research Department of Psychiatry, University of Minnesota, Minneapolis, MN USA

**Keywords:** Depression, Clinical pharmacology

## Abstract

The strategy of repeated ketamine in open-label and saline-control studies of treatment-resistant depression suggested greater antidepressant response beyond a single ketamine. However, consensus guideline stated the lack of evidence to support frequent ketamine administration. We compared the efficacy and safety of single vs. six repeated ketamine using midazolam as active placebo. Subjects received either six ketamine or five midazolam followed by a single ketamine during 12 days followed by up to 6-month post-treatment period. The primary end point was the change from baseline in the Montgomery-Åsberg Depression Rating Scale (MADRS) score at 24 h after the last infusion. Fifty-four subjects completed all six infusions. For the primary outcome measure, there was no significant difference in change of MADRS scores between six ketamine group and single ketamine group at 24 h post-last infusion. Repeated ketamine showed greater antidepressant efficacy compared to midazolam after five infusions before receiving single ketamine infusion. Remission and response favored the six ketamine after infusion 4 and 5, respectively, compared to midazolam before receiving single ketamine infusion. For those who responded, the median time-to-relapse was nominally but not statistically different (2 and 6 weeks for the single and six ketamine group, respectively). Repeated infusions were relatively well-tolerated. Repeated ketamine showed greater antidepressant efficacy to midazolam after five infusions but fell short of significance when compared to add-on single ketamine to midazolam at the end of 2 weeks. Increasing knowledge on the mechanism of ketamine should drive future studies on the optimal balance of dosing ketamine for maximum antidepressant efficacy with minimum exposure.

## Introduction

Ketamine, a glutamate receptor–blocking drug approved by the U.S. Food and Drug Administration for anesthetic use, has become a target of research for its antidepressant effects, and possible anti-suicidal effects. Single ketamine at subanesthetic dose of 0.5 mg/kg for 40 min has demonstrated improvement in mood within a few hours in some patients with treatment-resistant depression (TRD)^1,2^. The peak antidepressant effect occurs at 24-h post-infusion with gradual loss of therapeutic benefit between 5 and 8 days^1,3^. The strategy of repeated ketamine^4–7^ suggested that several infusions beyond a single ketamine increase antidepressant response and prolong its durability; however, open-label study designs or the use of saline as placebo limited more definite conclusions. We aimed to overcome these limitations by conducting a two-arm, randomized, active placebo-controlled trial. Through a total of six infusions in 12 days, we compared the efficacy and safety of six IV ketamine versus a single IV ketamine among patients with TRD. To balance the number of infusions, subjects in the single ketamine arm had five IV midazolam, a short-acting benzodiazepine and anesthetic agent, with fast onset of action, short elimination half-life, and similar time course of dissociative and nonspecific behavioral effects (e.g., sedation, disorientation) to ketamine^8^.

## Methods

### Study design and patients

The study was conducted at the Minneapolis Veterans Affairs Medical Center between April 2015 and March 2019. Subjects were outpatient, aged 18–75 years, met criteria for major depressive disorder (MDD) by the Structured Clinical Interview for DSM-IV(SCID), and had lack of response to at least two adequate antidepressant trials of different pharmacological classes during current major depressive episode (MDE) according to the Antidepressant Treatment History Form (ATHF)^9^. Systematic evaluation of previous antidepressants was assessed on all available information including VA pharmacy, and community-based records. In addition, participants had a score ≥32 on the Inventory of Depressive Symptomatology—Clinician Rated (IDS-C30)^10^ for severity of MDE at screening. Current antidepressant dosages including augmenting agents and/or frequency and duration of psychotherapy sessions remained stable for ≥6 weeks prior to consent and throughout the study.

Patient were excluded if they had a lifetime DSM-IV criteria for post-traumatic stress disorder (PTSD), mild to moderate traumatic brain injury, psychosis-related disorder, bipolar disorder, or any Axis I disorder other than MDD as the primary presenting problem. Patients with a history of alcohol or substance use disorder within 6 months of screening, imminent risk of suicidal/homicidal ideation and/or behavior with intent, a Mini-Mental State Examination score ≤27, positive urine toxicology or pregnancy test were also excluded. Medical records and laboratory tests were reviewed for any unstable medical illness. Study anesthesiologist were consulted on those cases with possible medical contraindication to participate.

### Study procedures

Randomization was conducted using permutated blocks of 4 and 1:1 assignment between treatments. The patients received six infusions on a Monday-Wednesday-Friday schedule over a 12-day period. Patients received five midazolam infusions followed by a single ketamine or six ketamine treatments (see Fig. SF1). The fact that the last infusion consisted of ketamine for both arms was kept undisclosed to subjects and raters of antidepressant outcomes at 24 h. Each infusion of ketamine at 0.5 mg/kg or midazolam at 0.045 mg/kg lasted 40 min. The selection of ketamine and midazolam doses was based on previous RCTs^1,11^. The dose of ketamine and midazolam were calculated by ideal body weight based on sex, age, height, and body frame in the Metropolitan Life Insurance tables. Group assignments for each participant was concealed in sequentially numbered, sealed, opaque envelopes with drug identity by the research pharmacist; investigators, raters, and patients were masked to treatment assignment.

Patients arrived at the infusion unit after fasting for at least 8 h. An indwelling catheter was placed in peripheral vein of non-dominant arm for medication administration; monitor of pulse, blood pressure, digital pulse oximetry, and respiratory rate was recorded every 10 min for 1 h beginning 10 min before infusion. Trained raters administered pre-infusion rating scales and repeated them again during a 2-h post-infusion monitoring period. A study physician was present throughout the infusion and study anesthesiologist (J.W.) was reached, if necessary. Medications such as labetalol, hydralazine, ondansetron, and flumenazil as well as a crash cart were available to manage adverse side effects. Guidelines established for clinically significant changes in vital signs and mental status during the ketamine infusions were as follows: systolic blood pressure >161 or <89, diastolic BP > 110 or <40; heart rate <40 or >130 beats/min; respiratory rate <10 or >30 per minute; pulse oximetry <90%; severe hallucinations, confusion, delusions, irrational behavior, or agitation. Before leaving the infusion unit, subjects demonstrated that all clinically significant side effects were resolved by a score ≥9 in the modified Aldrete scoring system^12^. Written instructions about potential side effects of sedatives and several measures to improve recovery at home (e.g., proper hydration and rest; abstain from alcohol) were provided at discharge to patient and companion adult.

### Outcomes

The study assessments were performed at days 0, 1, 3, 5, 8, 10, and 12 to assess the safety and efficacy of ketamine compared to active placebo during infusion phase. Post-treatment assessments were measured at weekly intervals for the first 4 weeks, at 2-week intervals for the next 8 weeks, and at 4-week intervals for the remaining 12 weeks. The primary outcome was change in depression severity measured by the clinician-administered Montgomery–Åsberg Depression Rating Scale score (MADRS)^13^ at 24 h (T_+24_) after the last infusion. Raters were trained in MADRS prior to and during study with a level of interrater reliability of 0.92. Secondary outcomes included change in MADRS score over time, rates of response (≥50% MADRS reduction in the baseline score), rates of remission (MADRS < 10), rates of response and remission over time, and durability of response for up to 6 months. Other secondary outcomes included clinical global impression (CGI) severity and improvement measures^14^, self-reported numeric rating scale (NRS) for pain^15^, Beck anxiety inventory (BAI)^16^, and credibility and expectancy questionnaire (CEQ)^17^ for clinical treatment. Cognitive performance (CogState)^18^, and functional neuroimaging on a sub-sample of patients will be reported separately.

General side effects were measured by the patient rated inventory of side effects (PRISE). Psychotogenic effects were measured with the four-item positive symptom subscale of the Brief Psychiatric Rating Scale (BPRS+)^19^ consisting of suspiciousness, hallucinations, unusual thought content, and conceptual disorganization; dissociative effects and manic symptoms were measured with the Clinician-Administered Dissociative States Scale (CADSS)^20^ and Young Mania Rating Scale (YMRS)^21^, respectively. Additionally, The Columbia-Suicide Severity Rating Scale (C-SSRS) Screening Version–Since Last Visit^22^ was rated during and after infusion phase for safety purposes.

### Statistical analysis

The power analysis was performed for MADRS score under the following assumptions: (1) analyses of covariance with the main effects of treatment (a single versus six infusions) and time (0 and Day 13), and the treatment by time interaction; (2) compound symmetric covariance matrix, and (3) 5% significance level. Our previous open-label study of six repeated ketamine in TRD patients^4^ showed a difference of 23 points in MADRS score from baseline to 24 h after the last infusion (mean MADRS score = 29.9, SD = 2.3 to mean MADRS score = 7.0, SD = 2.3). Based on these results, we estimated that a sample size of 21 patients per group was powered to detect a 10-point difference in change of MADRS score between the single versus repeated ketamine group. Repeated measures ANOVA was used to test for differences between groups in change from baseline to T_+24_ after the last infusion and baseline and T_+24_ after the fifth infusion. Multi-level models were used to compare groups on change across all MADRS measures. All tests were two-sided with significance at *p* < 0.05.

### Ethics approval

The authors assert that all procedures contributing to this work comply with the ethical standards of the relevant national and institutional committees on human experimentation and with the Helsinki Declaration of 1975, as revised in 2008. All procedures involving human subjects/patients were approved by Minneapolis VA Health Care System Institutional Review Board (protocol number 4533-B).

## Results

### Study participants

One-hundred seventy-eight individuals were pre-screened for eligibility through consultation with study physicians. Of these, 62 signed consent forms and underwent formal in-person screening (see the CONSORT diagram in Fig. SF2). There were four participants who failed screening; 58 subjects were randomized to treatment with four patients dropping prior to baseline data collection. All 54 subjects with baseline assessment, initiated study interventions and completed primary outcome end point. Five midazolam plus single ketamine group was composed by 29 subjects; a total of 25 subjects were in the six-ketamine group.

Participants’ baseline characteristics are summarized in Table 1. There was no significant difference between groups for demographic and clinical characteristics. The sample was mostly composed by middle aged, unemployed or retired, married, white males with a history of mood disorder among first-degree relatives. Patients had also a chronic history of depression that included at least one past psychiatric hospitalization with almost half of the patients reporting a previous suicidal attempt. Overall, patients had a current MDE with severe symptoms that failed to more than 02 adequate antidepressant trials and augmenting agents.

**Table 1:** Baseline demographic and clinical characteristics of patients with treatment-resistant depression treated with five midazolam plus a single ketamine versus six ketamine infusions.

Characteristics	Six ketamine		Five midazolam plus single ketamine		Overall sample	
	(*N* = 25)		(*N* = 29)		(*N* = 54)	
	Mean	SD	Mean	SD	Mean	SD
Age (years)	54.4	13.8	51.2	12.5	52.7	13.1
Level of education (years)	14.5	2.1	14.7	2.0	14.6	2.0
Age of first MDE (years)	25.6	11.6	25.0	10.1	25.3	10.4
Number of lifetime MDE	5.3	2.6	6.1	3.8	5.7	3.3
Duration of current MDE (weeks)	78.3	39.6	84.9	35.3	81.9	37.1
Number of adequate antidepressant trials during current MDE	2.2	0.4	2.4	1.1	2.3	0.8
Lifetime number of antidepressant trials	4.6	1.9	4.5	2.0	4.5	1.9
IDS-C	36.8	3.9	39.8	7.3	38.4	6.1
	N	%	N	%	N	%
Male	22	88.0	24	82.8	46	85.2
Race						
White	21	84.0	25	86.2	46	85.2
Black/African American	3	12.0	1	3.4	4	7.4
Other	1	4.0	3	10.3	4	7.4
Married	17	68.0	12	41.4	29	53.7
Unemployed/retired	16	64.0	19	65.5	35	64.8
Melancholic depression	8	32.0	6	20.7	14	25.9
Comorbid conditions						
Anxiety spectrum	4	17.4	5	20.0	9	16.7
Dysthymia	6	24.0	5	17.2	11	20.4
Sub-syndromal PTSD	6	24.0	5	17.2	11	20.4
Personality disorder	4	16.0	2	6.9	6	11.1
Chronic pain	10	40.0	9	31.0	19	35.2
Sleep disorder	14	56.0	14	48.3	28	51.9
Tobacco disorder	22	88.0	23	79.3	51	94.4
Severe MDE	15	60.0	11	37.9	26	48.1
History of AUD/SUD	10	40.0	8	27.6	18	33.3
Family history of						
Mood disorder	19	76.0	18	62.1	37	68.6
Other psychiatric disorder	3	12.0	7	24.1	10	18.5
AUD/SUD	9	36.0	11	37.9	20	37.0
Previous suicidal attempt	12	48.0	14	48.3	26	48.1
Previous psychiatric hospitalization	17	58.6	18	72.0	35	64.8
Past ECT treatment	4	16.0	7	24.1	11	20.4
Concurrent use of						
Augmenting agents	18	72.0	21	72.4	39	72.2
Benzodiazepines	12	48.0	11	37.9	23	42.6
Psychotherapy	9	36.0	12	41.4	21	38.9

### Primary outcome

**Fig. 1:**
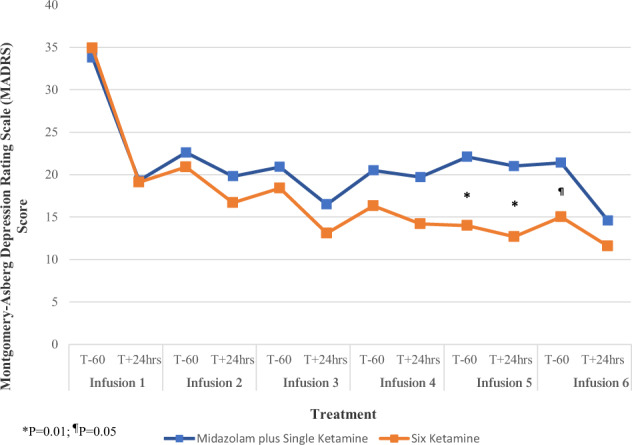
Mean Change in Severity of Depression between Six Ketamine versus Midazolam plus Single Ketamine Treatment within a 12-day Period. Severity of depression scores measured by the Montgomery-Åsberg Depression Rating Scale (MADRS) over 12 days between six ketamine and five midazolam plus single ketamine (last infusion) treatment groups in a randomized controlled trial in treatment-resistant major depression. Figure depicts mean MADRS scores in patients with treatment-resistant depression before and after 24 h of each ketamine (0.5 mg/kg) or single ketamine (0.5 mg/kg) preceded by five midazolam (0.045 mg/kg) infusion. * indicates significant difference in MADRS score between treatment condition (*p* = 0.01) before and at 24 h after infusion 5. ^¶^Indicates significant difference in MADRS score between treatment conditions (*p* = 0.05) before infusion 6.

There was no significant difference between single (MADRS mean change = 21.0, 95% CI = 17.2–24.8) versus six ketamine treatments (MADRS mean change = 17.2, 95% CI = 13.2–21.2) at T_+24_ after the end of treatment (six infusions) (*F*_1,52_ = 2.41, *P* = 0.13, ηp2 = 0.044) (Fig. 1). There was a significant change in MADRS score by groups over time when including all MADRS measures (*F*_11,541.11_ = 2.63, *P* = 0.003). The mean MADRS score among subjects receiving ketamine was significantly lower by 8.07 (95% CI, 1.67–14.46) prior to infusion 5 (*F*_1,95.7_ = 6.28, *P* = 0.014), by 8.29 (95% CI, 1.87–14.70) at T_+24_ after infusion 5 (*F*_1,96.7_ = 6.58, *P* = 0.012) and by 6.40 (95% CI, 0.01–12.79) prior to infusion 6 (*F*_1,95.7_ = 3.94, *P* = 0.050) compared to subjects receiving midazolam.

### Secondary outcomes

#### Response and remission rates

**Fig. 2:**
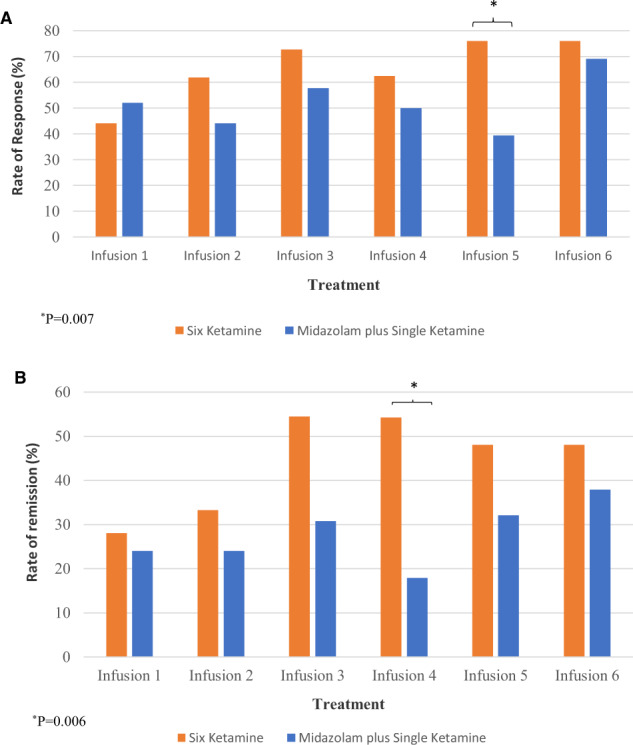
Antidepressant Response and Remission Rates between Six Ketamine versus Midazolam plus Single Ketamine Treatment within a 12-day Period. **a** Response rates over 12 days between six ketamine and five midazolam plus single ketamine (last infusion) treatment groups in a randomized controlled trial among subjects with treatment-resistant major depression. Response is defined as  > 50% decrease from baseline score measured by the Montgomery-Åsberg Depression Rating Scale. Figure depicts response in patients with treatment-resistant depression measured at 24h after each ketamine (0.5 mg/kg) or single ketamine (0.5 mg/kg) preceded by five midazolam (0.045 mg/kg) infusion. * indicates significant difference in response between treatment conditions (*p* = 0.007) at 24h after infusion 5 (Fig. 2b). Remission rates over 12 days between six ketamine and five midazolam plus single ketamine (last infusion) treatment groups in a randomized controlled trial among subjects with treatment-resistant major depression. Remission is defined as score < 10 in the Montgomery-Åsberg Depression Rating Scale. Figure depicts remission in patients with treatment-resistant depression measured at 24 h after each ketamine (0.5 mg/kg) or single ketamine (0.5 mg/kg) preceded by five midazolam (0.04 5mg/kg) infusion. * indicates significant difference in remission between treatment conditions (*p* = 0.006) at 24 h after infusion 4.

The response rates were not significantly different between groups at the end of six infusions (*X*^*2*^_1df_ = 0.73, *P* = 0.39) (Fig. 2a). There was also no significant difference in remission rates at T_+24_ after the last infusion between repeated midazolam plus single ketamine versus repeated ketamine (*X*^*2*^_1df_ = 0.56, *P* = 0.46) (Fig. 2b). Throughout the infusions phase, there was a significant difference in remission rates at T_+24_ after infusion 4 (six ketamine = 54.2% versus midazolam = 17.9%; *X*^*2*^_1df_ = 7.53, *P* = 0.006) and in response rates at T_+24_ after infusion 5 (six ketamine = 76% versus midazolam = 39.3%; *X*^*2*^_1df_ = 7.25, *P* = 0.007).

#### Other secondary outcomes

There was non-significant difference in anxiety as measured by BAI score change over times between groups at the end of infusion 5 (*F*_4,182.28_ = 1.74, *P* = 0.14) and infusion 6 (*F*_5,234.50_ = 1.53, *P* = 0.18). Self-rated pain also did not show significant difference between groups over infusion time points (*F*_5,238.34_ = 1.29, *P* = 0.27). There was a significant improvement in CGI for subjects in both groups, midazolam plus single ketamine (baseline mean = 5.14, 95% CI = 4.75–5.53 to post-infusion mean = 2.63, 95% CI = 2.14–3.12) (*t* (56) = 8.01, *p* < 0.001) and six ketamine (baseline mean = 5.32, 95% CI = 4.90–5.74 to post-infusion mean = 2.01, 95% CI = 1.55–2.48) (*t* (48) = 10.63, *p* < 0.001) without significant difference between groups (*F*_1,54.83_ = 3.59, *P* = 0.06) at T_+24_ after the end of treatment. There was no significant difference between groups in credibility (*F*_1,52_ = 0.19, *P* = 0.66) and expectancy (*F*_1,52_ = 0.03, *P* = 0.86) of treatment as measured by CEQ scores. There was no significant difference in the mean change of MADRS score from baseline to T_+24_ after the last infusion between subjects taking benzodiazepines (*N* = 11, mean change MADRS score = 22.9, SD = 9.5) or not taking benzodiazepines (*N* = 13, mean change in MADRS score = 23.2, SD = 6.5) (F_1,22_ = 0.01, *P* = 0.94) in the six ketamine group.

#### Durability of response

**Fig. 3:**
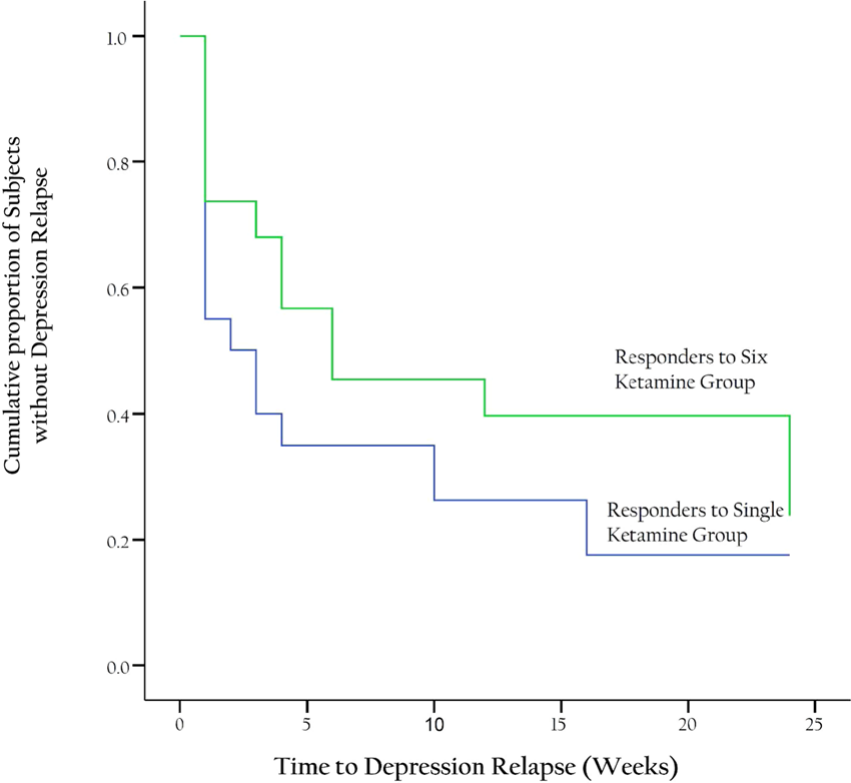
Time-to-relapse between responders to single vs six ketamine infusions in treatment-resistant depression. Figure depicts Kaplan–Meier analysis of responders for up 6 months of follow-up. Relapse was defined as <50% improvement in Montgomery–Åsberg Depression Rating Scale score at that visit compared with baseline.

MADRS scores at weekly for 4 weeks, biweekly for 8 weeks, and monthly for 3 months were used to determine time-to-relapse (MADRS > 50% from baseline) among patients who achieved response after the last infusion (*N* = 20 for midazolam plus single ketamine and *N* = 19 for six ketamine). The Kaplan–Meier estimates of the 6-month relapse rates after response (Fig. 3) was 75% (95% CI = 54.2–95.8%) and 68.4% (95% CI = 45.4–91.4%) for midazolam plus single ketamine and six ketamine group, respectively. The long-rank test revealed a non-statistically significant difference between relapse rates over time (*X*^*2*^_1df_ = 1.61; *P* = 0.21). The median time-to- relapse was 2 weeks for the midazolam plus single ketamine group, and 6 weeks for the six-ketamine group (95% CI for the hazard ratio = 0.3 to 1.4; *P* = 0.27).

### Adverse events

During infusion phase, the most common side effects for ketamine were general malaise (28.0%), decreased energy (28.0%), increased in blood pressure (24.0%), headaches (24.0%), fatigue (24.0%), nausea/vomiting (20.0%), anxiety (20.0%), and poor concentration (20.0%). Within the same period, the most common adverse events for midazolam plus single ketamine were anxiety (24.1%), decreased energy (17.2%), increased in blood pressure (17.2%), fatigue (13.8%), and headaches (13.8%).

#### Vital signs

There were 25 and 17 cases of mild and moderate adverse events during infusion phase, respectively. Moderate side effects were related to transient hypertensive episode (systolic > 161 or diastolic > 110 per protocol) that require at least one dose of antihypertensive medication such as labetalol or hydralazine as recommended by study anesthesiologist. Moderate adverse events also included nausea that required IV medication (e.g., ondansetron). Mild cases included increased blood pressure or nausea that subsided without medications, and IV site bruising but not pain. During infusions 1–5, there was a significantly higher systolic and diastolic blood pressure at T_+40m_ (systolic mean difference = 25.6, 95% CI: 16.4–34.9; diastolic mean difference = 12.9, 95% CI: 7.2–18.8) and diastolic blood pressure at T_+100m_ (mean difference = 12.9, 95% CI: 3.6–22.2) for the ketamine group compared to midazolam. There was no significant difference in heart rate between groups at any time point during infusions (data not shown).

#### Dissociative, psychomimetic, and mania

During infusions 1–5, there was a significantly greater dissociation measured by CADSS score at T_+40m_ (mean difference = 12.8, 95% CI: 10.1–15.6) for the ketamine group (mean = 15.3 95% CI: 13.3) compared to midazolam (mean = 2.5 95% CI: 0.6–4.4) (see Fig. SF3). All patients receiving ketamine had complete resolution of dissociative side effects within the 2-h monitoring period. Participants’ dissociative side effects (change in CADSS score a T_+40m_) were non-significantly correlated with antidepressant response (change in MADRS score) at T_+24h_ post-infusion for midazolam (Pearson’s *r* = –0.03, *p* = 0.89) or ketamine (Pearson’s *r* = 0.04, *p* = 0.86). Comparison of change in CADSS scores in the ketamine group at T_+40m_ (peak of side effects) revealed non-significant change in dissociative side effects with repeated infusions (*F*_5,220.28_ = 1.83, *P* = 0.11). There was not significant change within or between groups regarding psychotic (BPRS+) (see Fig. SF4) or elevated mood symptoms (YMRS-item 1) at any time point throughout infusions.

#### Serious adverse events

There were two cases deemed as a serious adverse event that required IRB report (see Table ST1): one patient (assigned to midazolam) complained of headaches triggered by loud noises and bright lights after post-infusion MRI as part of study protocol; another patient (assigned to ketamine) reported mild headaches during second infusion, which increased during follow-up phase. Headaches in both cases were considered unrelated to study drugs and eventually subsided. Through interviews with subjects, no suicidal attempts nor drug-seeking behavior were elicited throughout the study.

#### Blinding

Subjects were asked prior to and at the end of the last infusions (T_+160_) about treatment allocation. Prior to the last infusion (midazolam versus ketamine), 6.3% among midazolam cases and 56.3% among ketamine cases were erroneous about what treatment they were assigned to (*X*^*2*^_1df_ = 9.30; *P* = 0.002). At the end of the last infusion (midazolam plus single ketamine versus six ketamine), 10.7% among midazolam cases and 50.0% among ketamine cases guessed incorrectly about assigned treatment (*X*^*2*^_1df_ = 9.12; *P* = 0.002). Raters, who were different during infusions days from those rating antidepressant outcomes at 24 h., incorrectly guessed 20.8% of midazolam cases and 7.1% of repeated ketamine cases prior to the last infusion (*X*^*2*^_1df_ = 1.25; *P* = 0.26). After the last infusion, 6.9 and 26% raters were incorrect about treatment assignment among midazolam plus single ketamine and repeated ketamine cases, respectively (*X*^*2*^_1df_ = 3.62; *P* = 0.06).

## Discussion

This single center study of Veterans with moderate-to-severe, recurrent, TRD showed that for the primary outcome measure, there was no significant difference in change of MADRS scores between groups at 24 h post-last infusion at the end of 12 days of treatment. Repeated ketamine showed greater antidepressant efficacy compared to midazolam after five infusions before receiving single ketamine as the last infusion. The rate of remission and response was greater among subjects in the six-ketamine group after infusion 4 and 5, respectively. For those subjects who responded to either assigned treatment, six ketamine tripled the median time-to-relapse over a 6-month period as compared to those in the midazolam plus single ketamine group; however, it fell short of statistically significant difference. Transient psychoactive and hemodynamic effects during ketamine infusion were consistent with those in previous reports^7,6^ and did not increase when repeated treatments were given in a short-term fashion.

The prospect of whether repeated dosing of ketamine offer safe and superior antidepressant outcomes is a critical question as studies have shown that frequent use of ketamine could lead to cognitive impairments^23^, dissociation, and poor impulse control^24^. Medical, legal, and even ethical concerns of using repeated ketamine to treat psychiatric conditions have been raised^25^. Despite the small sample in this study, this is the largest randomized study that compare repeated ketamine versus an active placebo in TRD. Previously, Singh and colleagues^26^ showed that both twice-weekly and thrice-weekly administration of ketamine over 15 days of treatment had greater antidepressant efficacy versus saline (least-square mean change in MADRS score of 16.0 and 16.4 points, respectively). An open-label study of thrice ketamine weekly for 2 weeks among unipolar and bipolar depressed Chinese patients^7^ showed an overall reduction of 15.5 and 18.7 points in MADRS score at 24 h. after ketamine infusion 5 and 6, respectively. A more recently study among patients with TRD who responded and then relapsed after a cross-over single ketamine (versus midazolam)^6^ showed a reduction of 12 points in MADRS score after completing an open-label treatment of thrice a week for 2 weeks.

From these previous studies, our findings support the idea that repeated ketamine further reduce the severity of depression in TRD. However, the antidepressant improvement through six ketamine treatments was not significantly different when compared to a single ketamine. Previous reports on repeated ketamine consistently found that the largest improvement (≥50%) in TRD occurred after the first infusion^26,7,6,4,27,28^. It is likely then that the relatively small sample in our study was insufficient to capture any significant difference between antidepressant gain beyond the first infusion in the repeated ketamine group versus that in the single ketamine. On the other hand, subjects in the repeated ketamine group achieved a mean group treatment difference of −4.0 against repeated midazolam plus single ketamine at 24 h after the last infusion. A 4-point difference on the MADRS has been suggested as a stringent criterion for judging whether an effect size is clinically relevant^29^ with more recent reports suggesting a minimal clinically important difference threshold of 2 points in MADRS^30^. Moreover, Popova and colleagues^31^ on the recent approval of esketamine, the S-enantiomer of ketamine racemate, by the U.S. Food and Drug Administration for TRD in adults, reported a significant difference of four points at day 28 in the change of MADRS in a multicenter phase 3 RCT comparing esketamine plus antidepressant versus antidepressant plus placebo. Thus, we argue that despite the type II error in our study, there was a clinically significant difference supporting the use of repeated infusions.

Our study also support recent review of masking in ketamine studies for mood disorders^32^ namely that midazolam as a comparator yields smaller effects of ketamine than those which used saline. However, participants at the dose of midazolam used in this study (0.045 mg/kg) had lower intensity of dissociative symptoms as compared to ketamine, and in fact, correctly guessed treatment assignment in more than 90% of the cases even before exposure to ketamine at the last infusion. Therefore, while the use of an active placebo in repeated ketamine administration is encouraged to avoid overestimating ketamine’s antidepressant effect, an optimal comparator that mimic the dissociative and psychomimetic side effects of ketamine and preserve masking is still missing. As an alternative, low dose of ketamine such as 0.2 mg/kg^11,33^ have shown to lack antidepressant effect while still inducing dissociative effects.

In general, serial ketamine had greater side effects as compared to midazolam plus single ketamine. Acute, mild, and transient dissociation was the most common side effect reported on ketamine dosing days with other treatment emergent adverse events appeared similar in frequency as reported previously (e.g., increased in blood pressure, decreased energy, headache, nausea, etc.). The frequency of dissociative side effects did not appear to change with subsequent ketamine infusions and was not associated with antidepressant response. Although there have been reports of an association between dissociative side effects and antidepressant response to ketamine^34,35^, additional research is required to further explain whether dissociation account for antidepressant improvement.

We found that during the 6-month follow-up among those who responded to either assigned treatment after six infusions, multiple ketamine prolonged the response for a median of 6 weeks compared to 2 weeks of midazolam plus single ketamine. Considering that response to a single dose of ketamine tends to dissipate typically 2 weeks if ketamine was not repeated, an effective strategy to maintain response after cessation of infusions is critical. Phillips and colleagues^6^ demonstrated that four weekly administration of intravenous ketamine maintain response after an acute open-label ketamine treatment of thrice a week for 2 weeks. Similarly, patients who achieved stable remission and stable response after 16 weeks of initial treatment with esketamine, decreased the risk of relapse by 51% and 70%, respectively, during a maintenance phase of esketamine and antidepressant treatment compared to antidepressant and placebo treatment^36^. These findings support the possibility that repeated ketamine in combination to conventional antidepressants is a feasible strategy to maintain treatment response. Safety data and the optimal benefit-risk ratio for long-term treatment with ketamine is warranted.

The immediate and delayed antidepressant effects of ketamine, which are independent of its sustained blood concentrations, could be based on different mechanisms. Studies have focused on the non-competitive antagonist action on the NMDA receptor and subsequent activation of AMPA receptors driving acute antidepressant effect^37,38^. The modulation of glutamate receptors triggers downstream the modulation of synthesis and release of brain-derived neurotrophic factor and enhances synaptic plasticity via activation of molecular targets such as mammalian target of rapamycin and eukaryotic elongation factor 2. These systems may be involved in the delayed maintenance of response rather than the acute and rapid antidepressant effect of ketamine. Recent pre-clinical studies support this idea by showing that ketamine’s effects on behavior and ensemble activity occur rapidly and precede its effects on spine formation in the prefrontal cortex^39^. These newly formed spines are not required for inducing ketamine’s effects acutely but are critical for sustaining the antidepressant effect over time. More recently, preliminary clinical findings suggest a central role for opiate agonism in the antidepressant effects of ketamine^40^ although conflicting results were also reported^41^. Overall, a growing body of evidence indicates that additional mechanisms, not mutually exclusive and possibly complementing each other, are likely mediating the unique properties of ketamine and its ketamine metabolites [e.g., (2R,6R)-hydroxynorketamine] as antidepressant^42^.

### Limitations of the study

The study for the primary outcome was underpowered despite surpassing recruitment goal for a total of 54 participants. A total of 87 patients per group would have needed to detect the difference of 4 points in MADRS score between groups at end of six infusions (21 versus 17.2). The study design aimed to maximize recruitment while responding a pragmatic question on the efficacy of repeated ketamine. Ideally, a trial would have included an additional placebo-controlled arm composed exclusively by midazolam infusions. However, such a design would have been needed much more expensive, more problematic to recruit for, and the inclusion of a non-therapeutic intervention as control might have raised ethical issues. In the current study, we found a placebo response of nearly 40% by midazolam during the first infusion, which is within the range of 35–40% response rates in antidepressant trials^43^ but greater than previously reported in ketamine studies. For instance, Murrough and colleagues found a 28% response after a single midazolam infusion compared to 64% response to ketamine. As placebo response rates increases with the likelihood of receiving active treatment^44^, we can only speculate that the expectation to receive at least one ketamine treatment may have enhanced the active placebo effect of midazolam particularly during the first infusion. Remarkably, despite cumulative active placebo response by midazolam, a single ketamine as the last infusion provide further antidepressant gains and erased any statistical difference in favor of six ketamine up until that point.

The study also allowed concomitant psychiatric medication regimen on stable dosages for at least 6 weeks prior to study onset. While the intention was to recreate real-clinical settings with ketamine as an augmenting intervention, we cannot rule out the impact of concurrent medications. For instance, preliminary data reported that benzodiazepines could attenuate the response to ketamine^45^, a finding we did not replicate in this study. Because the study was conducted at a single VA site, results should be carefully extrapolated to other VA sites or non-Veterans. The population studied was predominantly male (85%) and older (average age of 53-years old), compared to study population in previous trials of TRD and ketamine in which younger participants (i.e., 43-years old^5^) and the number of women typically exceed the proportion of male participants. Clinical studies have not corroborated sex differences in ketamine response^46^. Another significant difference is that 20% of patients had sub-syndromal PTSD. While few and small studies have shown the efficacy of single^47^ and repeated ketamine^48^ in PTSD to be comparable with depression, it is unclear whether MDD and comorbid PTSD symptoms could have changed the treatment response to ketamine.

## Conclusion

While acute repeated ketamine showed greater antidepressant efficacy to midazolam after five infusions, this antidepressant difference fell short of significance when a single ketamine was added to the midazolam group as the last infusion. Larger studies would be needed to confirm or not whether repeated ketamine appears to prolong response compared to single ketamine. Increasing knowledge on the mechanism of ketamine should drive future studies on the optimal balance of dosing ketamine for maximum antidepressant efficacy with minimum exposure.

## Supplementary information

Supplemental Figure 1

Supplemental Figure 2

Supplemental Figure 3

Supplemental Figure 4

Supplemental table 1

CONSORT 2010 checklist of information to include when reporting a randomised trial

## Data Availability

The authors had full access to the data that were used in the current analysis.
