# Supplementary material for: A randomized, double-blind, active placebo-controlled study of efficacy, safety, and durability of repeated vs single subanesthetic ketamine for treatment-resistant depression
Source: Transl Psychiatry. 2020 Jun 26;10:206. doi: 10.1038/s41398-020-00897-0 (PMC7319954; doi:10.1038/s41398-020-00897-0)

Supplemental Figure 2. Participant Flow in a Study of Intravenous Ketamine in Treatment-Resistant Depression

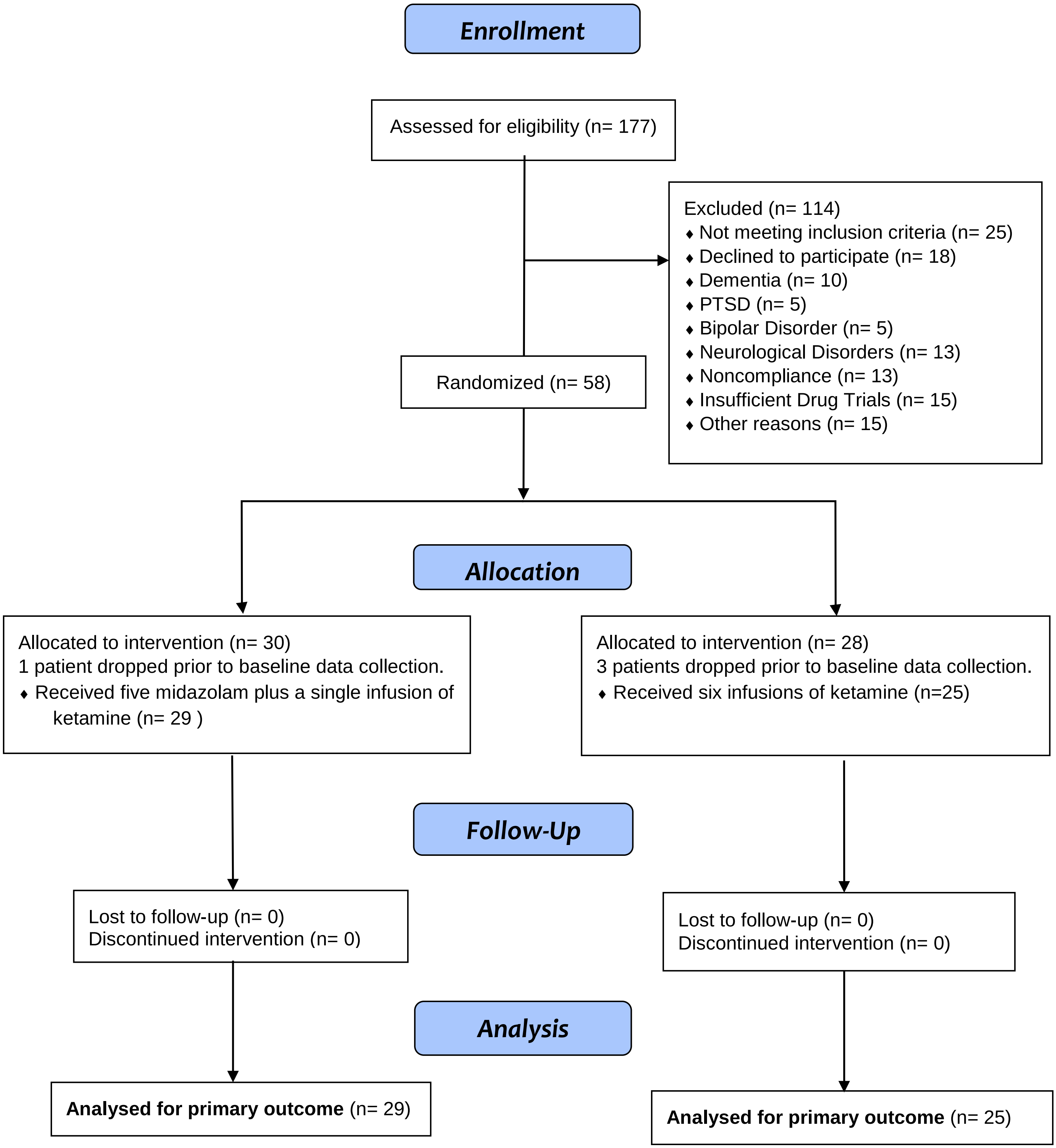

Supplement: Supplementary file 2 — Supplemental Figure 2 [file 41398_2020_897_MOESM2_ESM.pdf]
