# Supplementary material for: A randomized, double-blind, active placebo-controlled study of efficacy, safety, and durability of repeated vs single subanesthetic ketamine for treatment-resistant depression
Source: Transl Psychiatry. 2020 Jun 26;10:206. doi: 10.1038/s41398-020-00897-0 (PMC7319954; doi:10.1038/s41398-020-00897-0)
Supplement: Supplementary file 5 — Supplemental table 1 [file 41398_2020_897_MOESM5_ESM.doc]

**Supplemental Table 1. Participants with Serious Adverse Events (SAEs)**

| Patient | Phase of Study | Description of SAE | Intervention | Treatment Assignment and Relation |
| --- | --- | --- | --- | --- |
| KD-91 | Pre-infusion | During follow-up phase, patient complained that daily headaches triggered by loud noises and bright lights started after pre-infusion head MRI. Described as oppressing in the temples without dizziness, nausea, vomiting, or auras. Patient did not take any medication to relieve headaches. | Patient was reassured about lack of radiation involved in MRI. No contrast was used in MRI. Primary care physician was notified, and headaches eventually subsided without further intervention. | Midazolam plus single ketamine. None. |
| KD-104 | Infusion | After completing infusion phase, patient complained of headaches after second infusion, and deemed mild and intermittent. During follow-up, headaches became more frequent accompanied with nausea, blurry visions and sensitivity to lights and sounds. Denies previous migraine or chronic headaches. | Case was consulted with primary care physician, neurologist and study anesthesiologist. Head MRI and MRV became normal. Indomethacin was initiated. After two months of initial complaints, headaches eventually subsided. | Six ketamine. None. |
