# Supplementary material for: A randomized, double-blind, active placebo-controlled study of efficacy, safety, and durability of repeated vs single subanesthetic ketamine for treatment-resistant depression
Source: Transl Psychiatry. 2020 Jun 26;10:206. doi: 10.1038/s41398-020-00897-0 (PMC7319954; doi:10.1038/s41398-020-00897-0)
Supplement: Supplementary file 4 [file 41398_2020_897_MOESM1_ESM.doc]

Supplemental Figure 1

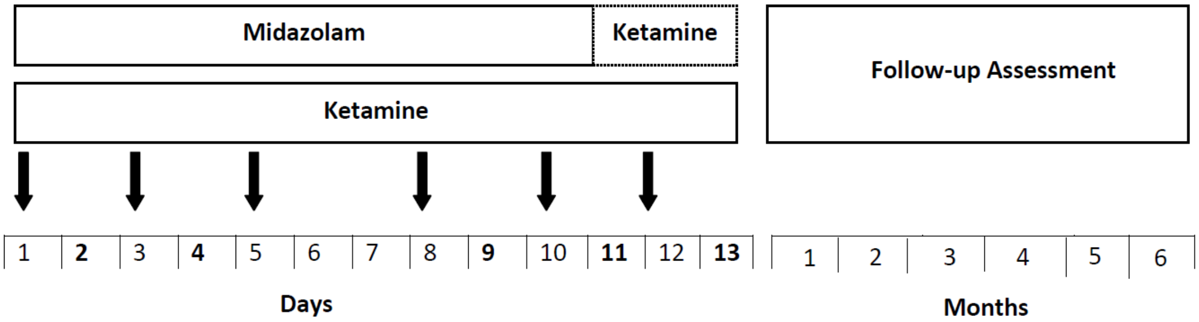

Supplemental Figure 1. Figure depicts treatment groups consistent of six infusions of ketamine 0.5 mg/kg versus five infusions of midazolam 0.045 mg/kg plus a single infusion of ketamine 0.5 mg/kg administered on a Monday-Wednesday-Friday schedule for 12 days. Arrows indicated days of infusions corresponding to study days 1, 3, 5, 8, 10, and 12. Depression severity is measured by the Montgomery–Åsberg Depression Rating Scale (MADRS) 24 hours after each infusion corresponding to days 2, 4, 6, 9, 11 and 13 (in bold). Follow-up period will last 6 months or until relapse is observed.
